# Discovery of a parallel family of euglenatide analogs in *Euglena gracilis*

**DOI:** 10.1007/s13659-024-00490-8

**Published:** 2025-01-06

**Authors:** Ahmed H. Elbanna, Xinhui Kou, Dilip V. Prajapati, Surasree Rakshit, Rebecca A. Butcher

**Affiliations:** 1 Department of Chemistry, University of Florida, Gainesville, FL 32611 USA; 2 Department of Pharmacognosy, Faculty of Pharmacy, Cairo University, Cairo, 11562 Egypt

**Keywords:** *Euglena gracilis*, Euglenatide, Natural products, Polyketide, Nonribosomal peptide

## Abstract

The euglenatides are a family of hybrid polyketide-nonribosomal peptides produced by the unicellular algae *Euglena gracilis*. These compounds have antiproliferative activity against fungal pathogens and mammalian cancer cell lines. Analysis of *E. gracilis* extracts revealed that the algae produce not only the euglenatides, but also a corresponding family of analogs that have the same molecular weights as the euglenatides, but are lacking the characteristic triene chromophore. In comparison to the euglenatides, the activity of these analogs is greatly reduced in a mammalian cytotoxicity assay, indicating that the triene is critical to the biological activity of the euglenatides.

**Graphical abstract:**

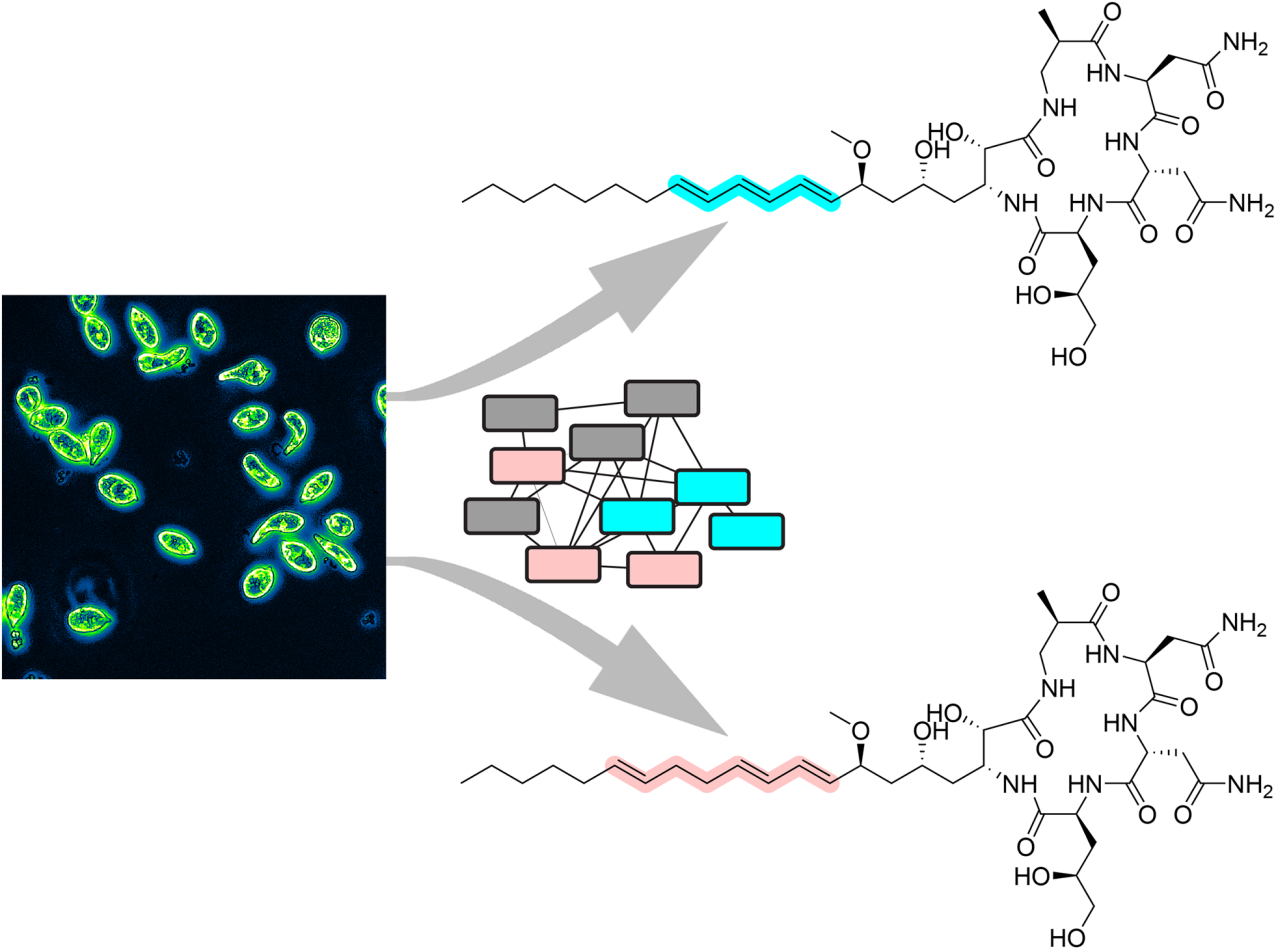

**Supplementary Information:**

The online version contains supplementary material available at 10.1007/s13659-024-00490-8.

## Introduction

Polyketides and nonribosomal peptides are complex natural products that are produced on large enzymatic assembly lines known as polyketide synthases (PKSs) and nonribosomal peptide synthetases (NRPSs), respectively [1, 2]. Many of these natural products have been developed into important therapeutics, and thus discovery of structurally novel polyketides and nonribosomal peptides from underexplored sources is of interest. One of these potential sources is the unicellular flagellate algae *Euglena gracilis*, which can grow through photoautotrophic growth, using light as energy, heterotrophic feeding, using an external carbon source as energy, or a mixture of the two growth modes [3]. Although *E. gracilis* has been studied extensively as a potential source of food and biofuel, relatively little is known about its natural product biosynthetic potential. Even though the genome of *E. gracilis* has been only partially sequenced, these efforts indicate that the genome includes a large number of PKSs and NRPSs and other enzymes involved in secondary metabolism [4, 5].

**Fig. 1:**
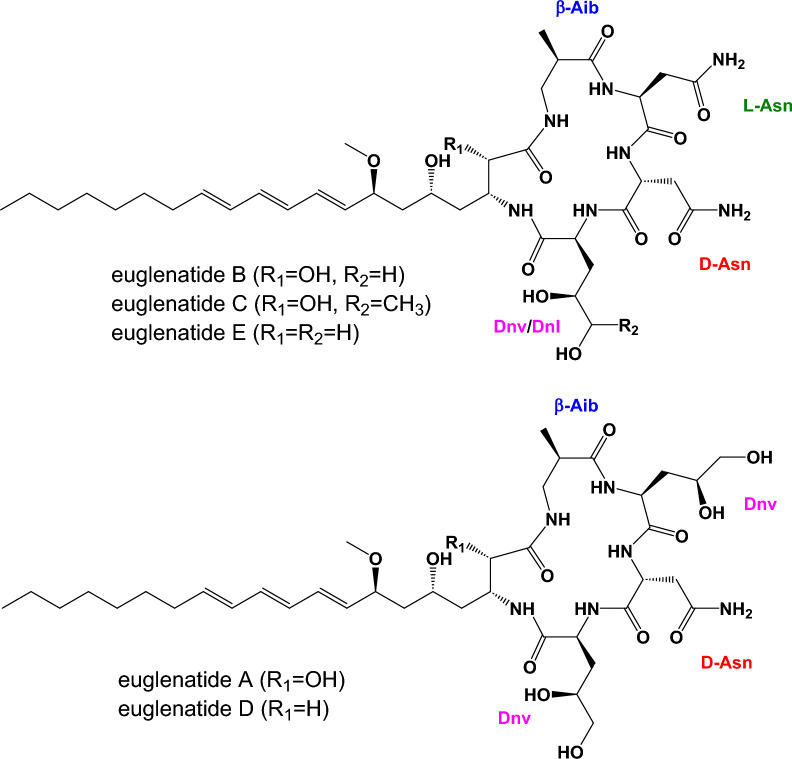
Previously reported structures of euglenatides A–E. The unusual amino acids found in the euglenatides include β-aminoisobutyric acid (β-Aib), 4,5-dihydroxynorvaline (Dnv), and 4,5-dihydroxynorleucine (Dnl)

Recently, *E. gracilis* was shown to produce a family of hybrid polyketide-nonribosomal peptides, euglenatides A–E, with euglenatide B being the most abundantly produced (Fig. 1) [6]. Interestingly, the euglenatides are structurally similar to a family of hybrid polyketide-nonribosomal peptides previously identified in the nematode *Caenorhabditis elegans* [7–9]. The euglenatides have antiproliferative activity against multiple cancer cell lines in the mid-nanomolar range and several fungal pathogens in the low micromolar range, but have no activity against bacteria [6]. A survey of additional *Euglena* species suggests that they also produce similar natural products. Interestingly, production of the eugenatides did not occur in *E. gracilis* grown in rich medium and only occurred in *E. gracilis* grown in minimal medium containing specific amino acids, either glutamate or asparagine, under photosynthetic conditions [6].

Here, in analyzing extracts from *E. gracilis*, we discovered a new, parallel family of analogs that are produced in roughly equivalent amounts to euglenatides A–C. In comparison to the euglenatides, these analogs have the same molecular weights. However, the triene that is present in the euglenatides is disrupted in the analogs in that one of the double bonds is shifted by two carbons. The analogs are tenfold less bioactive against A549 lung adenocarcinoma cells than the euglenatides, indicating that the triene motif is important for activity.

## Results and discussion

**Fig. 2:**
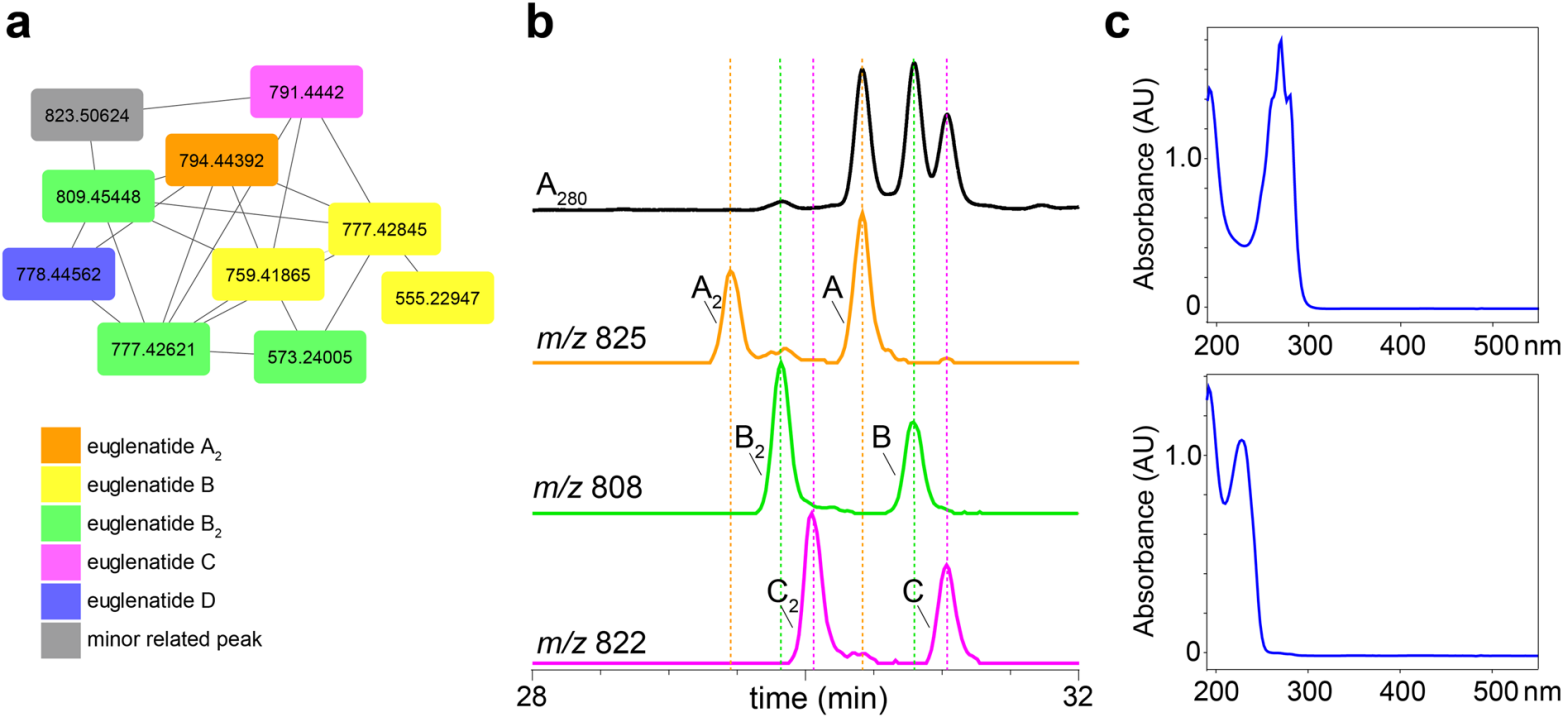
*E. gracilis* produces the euglenatide analogs, euglenatides A_2_, B_2_, and C_2_. **a** Molecular networking analysis of crude *E. gracilis* extracts analyzed in positive (ESI+) mode. The orange node represents the [M-CH_4_O + H]^+^ ion for A_2_, the yellow nodes represent the [M-CH_4_O + H]^+^, [M-CH_6_O_2_ + H]^+^, and fragment ions of B, the green nodes represent the [M + H]^+^, [M-CH_4_O + H]^+^ and fragment ions of B_2_, the pink node represents the [M-CH_4_O + H]^+^ ion of C, and the purple node represents the [M-CH_4_O + H]^+^ ion of D. **b** Extracted ion chromatograms (EICs) of euglenatides A–C and their analogs A_2_–C_2_ analyzed in negative (ESI-) mode, along with the absorbance at 280 nm. The fraction analyzed here has roughly equal amounts of the different euglenatides and their analogs. However, in the crude extracts, euglenatide B and B_2_ are much more abundant than euglenatides A, A_2_, C, and C_2_. **c** UV–Vis spectrum of euglenatide B (top) and euglenatide B_2_ (bottom)

**Fig. 3:**
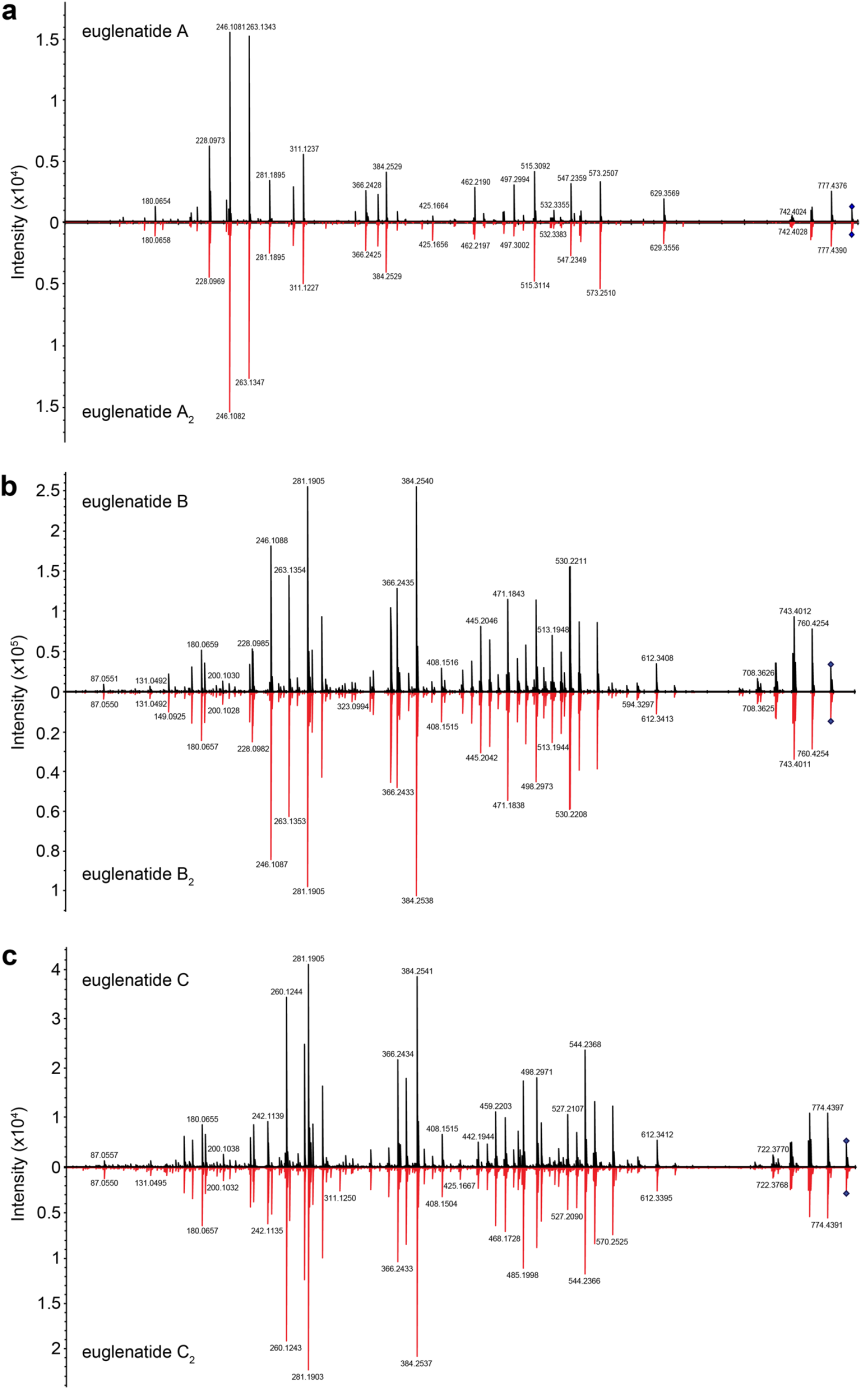
MS–MS spectra of the euglenatides and their analogs. Mirrored MS–MS spectra for euglenatides A and A_2_ (**a**), euglenatides B and B_2_ (**b**), euglenatides C and C_2_ (**c**)

In order to determine whether *E. gracilis* produces additional euglenatides or euglenatide biosynthetic intermediates, we cultured *E. gracilis* using similar methods to those reported by Aldholmi et al*.* [6]; that is, we used minimal medium in which the carbon source was sodium acetate and the nitrogen source was glutamate, and we cultured the algae under photosynthetic conditions. In our hands, euglenatide B was more than tenfold more abundant than euglenatides A and C, euglenatide D was only minorly present, and euglenatide E was not detectable. Using molecular networking and LC–MS analysis of *E. gracilis* crude extracts, we noted that *E. gracilis* produces not only the euglenatides, but also a corresponding set of euglenatide analogs that we named euglenatides A_2_-C_2_ (Fig. 2) [10, 11]. These analogs have the same exact masses and MS fragmentation patterns as euglenatides A-C (Fig. 3, Fig. S1), but are slightly more polar based on their retention times (Fig. 2b). Unlike euglenatides A–C, which have a UV–Vis chromophore characteristic of a conjugated triene, euglenatides A_2_–C_2_ do not absorb at 280 nm and do not possess a conjugated triene (Fig. 2b, c). Each euglenatide is produced at roughly similar levels as its corresponding euglenatide analog. We have not been able to enhance the production of the euglenatide analogs relative to the euglenatides by harvesting the *E. gracilis* culture at a particular time point or utilizing a particular nitrogen source (glutamate versus asparagine).

To further characterize the chemical structures of the euglenatide analogs, we purified the most abundant one, euglenatide B_2_, as well as euglenatide B for comparison purposes. Chromatographic fractionation of defatted methanolic extracts of *E. gracilis* by reversed phase C18 flash chromatography, LH-20 Sephadex chromatography, and semipreparative HPLC allowed the purification of the two euglenatides (Fig. S2). Analysis of the 1D and 2D NMR (DMSO-*d*_6_) data for euglenatide B (Table 1; Table S1 and Figs. S3, S4) in comparison to literature [6] confirmed its identification (Fig. 1).

**Table 1:** 1D and 2D NMR (DMSO-*d*_6_) data for euglenatide B and its analog euglenatide B_2_

No	Euglenatide B			Euglenatide B_2_		
	Type	^13^C ppm	^1^H ppm mult. (*J* in Hz)	Type	^13^C ppm	^1^H ppm mult. (*J* in Hz)
*Dnv*						
1	C	172.5	–	C	172.5	–
2	CH	50.3	4.30, ddd (11.0, 7.8, 2.8)	CH	50.3	4.29, ddd (11.0, 7.8, 2.8)
2-NH	NH	–	7.65, d (7.8)	NH	–	7.65, d (8.0)
3	CH_2_	34.3	1.82, ddd (14.0, 11.0, 2.8)	CH_2_	34.4	1.82, ddd (13.8, 10.7, 2.8)
			1.70, m			1.69, m
4	CH	67.5	3.44, m	CH	67.5	3.43, m
4-OH	OH	–	4.49, d (5.0)	OH	–	4.48, d (5.0)
5	CH_2_	66.3	3.31, m	CH_2_	66.3	3.31, m
			3.24, m			3.24, m
5-OH	OH	–	4.39, m	OH	–	4.39, m
*D-Asn*						
6	C	171.2	–	C	171.2	–
7	CH	49.7	4.51, m	CH	49.7	4.50, m
7-NH	NH	–	8.20, br d (7.8)	NH	–	8.13, br d (7.4)
8	CH_2_	36.2	2.93, dd (16.0, 7.0)	CH_2_	36.2	2.92, dd (16.0, 6.8)
			2.56, dd (16.0, 3.6)			2.55, dd (16.0, 3.7)
9	C	173.1	–	C	173.2	–
9-NH	NH_2_	–	7.87, s	NH_2_	–	7.88, s
			7.14, s			7.17, s
*L-Asn*						
10	C	169.7	–	C	169.7	–
11	CH	51.6	4.04, m	CH	51.6	4.04, ddd (9.8, 6.4, 4.1)
11-NH	NH	–	9.05, d (6.4)	NH	–	9.04, d (6.7)
12	CH_2_	35.2	2.86, dd (15.8, 4.0)	CH_2_	35.2	2.85, dd (15.6, 4.0)
			2.43, dd (15.8, 9.3)			2.43, dd (15.6, 9.3)
13	C	172.2	–	C	172.1	–
13-NH	NH_2_	–	7.37, s	NH_2_	–	7.37, s
			6.86, s			6.86, s
*β-Aib*						
14	C	176.4	–	C	176.4	–
15	CH	37.6	2.66, m	CH	37.6	2.65, (9.8, 7.1, 2.6)
16	CH_2_	41.7	3.23, m	CH_2_	41.7	3.23, m
			2.99, br d (13.2)			2.99, br d (13.2)
16-NH	NH	–	7.72, br s	NH	–	7.61, br s
17	CH_3_	17.2	1.03, d (7.0)	CH_3_	17.1	1.03, d (7.0)
*Polyketide*						
18	C	172.1	–	C	172.2	–
19	CH	72.6	3.89, m	CH	72.6	3.89, m
19-OH	OH	–	5.10, br s	OH	–	5.10, very broad
20	CH	49.1	3.88, m	CH	49.1	3.88, m
20-NH	NH	–	6.93, d (9.1)	NH	–	6.92, d (8.9)
21	CH_2_	40.5	1.69, m	CH_2_	40.5	1.68, m
			1.19, m			1.18, m
22	CH	62.9	3.49, m	CH	62.9	3.49, m
22-OH	OH	–	4.38, m	OH	–	4.36, m
23	CH_2_	43.7	1.47, m	CH_2_	43.8	1.46, m
			1.33, m			1.31, m
24	CH	77.8	3.72, ddd (11.5, 7.6, 3.7)	CH	77.8	3.7, ddd (11.5, 8.1, 3.5)
25	CH	133.9	5.50, dd (14.6, 7.6)	CH	132.2	5.40, m
26	CH	131.2	6.19, dd (14.6, 10.4)	CH	131.3	6.12, dd (15.0, 10.4)
27	CH	130.0	6.14, dd (14.6, 10.4)	CH	129.9	6.04, dd (15.0, 10.4)
28	CH	132.8	6.23, dd (14.5, 10.4)	CH	133.8	5.69, dt (14.5, 6.5)
29	CH	130.3	6.06, dd (15.0, 10.4)	CH_2_	32.2	2.10, m
30	CH	135.1	5.71, dt (15.0, 7.1)	CH_2_	31.7	2.04, m
31	CH_2_	32.1	2.06, q (7.0)	CH	129.3	5.38, m (*16.2, 5.4*)^a^
32	CH_2_	28.7	1.34, m	CH	130.5	5.40, m (16.2, 5.4)^a^
33	CH_2_	28.50	1.25, m	CH_2_	31.9	1.93, q (6.1)
34	CH_2_	28.52	1.25, m	CH_2_	28.6	1.30, m
35	CH_2_	31.2	1.24, m	CH_2_	30.7	1.22, m
36	CH_2_	22.0	1.26, m	CH_2_	21.9	1.25, m
37	CH_3_	13.9	0.85, t (7.0)	CH_3_	13.9	0.85, t (7.0)
38	OCH_3_	55.5	3.13, s	OCH_3_	55.5	3.11, s

^a^*J* values were obtained from DQF-COSY data

**Fig. 4:**
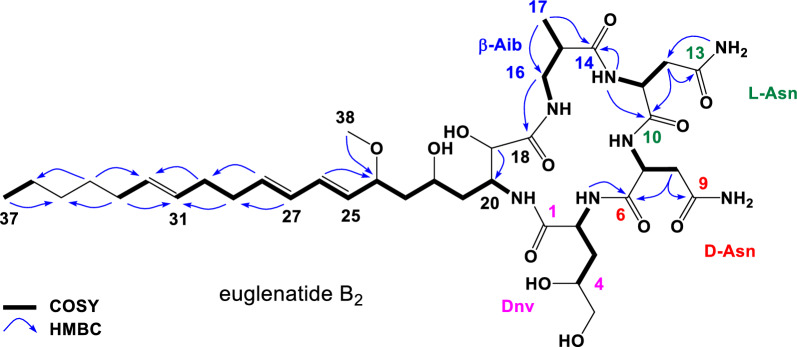
Key 2D NMR correlations for the euglenatide B analog, euglenatide B_2_

**Fig. 5:**
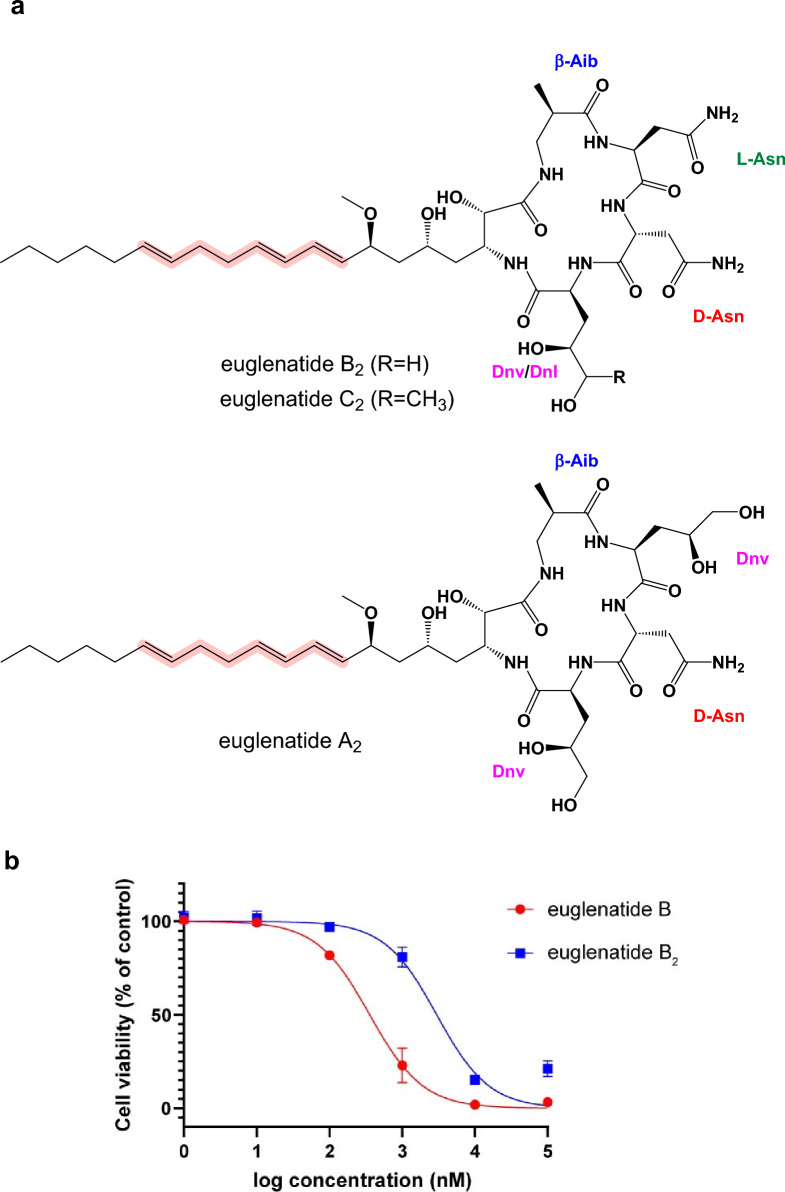
Chemical structures of euglenatides A_2_, B_2_, and C_2_ (**a**) and the antiproliferative activity of euglenatides B and B_2_ against A549 cells (**b**)

Comparison of the 1D NMR (DMSO-*d*_6_) data for euglenatide B_2_ with that of euglenatide B disclosed common peptide and polyketide substructures, with the significant differences being the presence of two extra allylic methylene resonances in euglenatide B_2_ at C-29 (*δ*_2H_ 2.10, m; *δ*_C_ 32.2) and C-30 (*δ*_2H_ 2.04, m; *δ*_C_ 31.7), accompanied by a change in the pattern of olefinic methine resonances (Fig. 4, Table 1; Table S2, and Figs. S5–S13). These NMR changes in euglenatide B_2_ suggested that one double bond (Δ^31^) is separated from the diene (Δ^25^ and Δ^27^) by two methylenes at C-29 and C-30, which explains the absence of the conjugated triene chromophore in the UV–Vis spectrum. Diagnostic 2D NMR correlations (shown in Fig. 4) confirmed the double bond shift and the molecular connectivity of euglenatide B_2_, while NMR similarities with euglenatide B, including comparable *J* values, and biosynthetic considerations suggested a common absolute configuration. Finally, Double Quantum Filtered (DQF)-COSY NMR analysis of euglenatide B_2_ allowed the assignment of the Δ^31^ double bond geometry as *E* (trans) based on a coupling constant (*J*_*31,32*_) of 16.2 Hz (Figs. S10, S11). This assignment is further supported with the upfield chemical shifts of the allylic methylenes at C-30 (*δ*_C_ 31.7) and C-33 (*δ*_C_ 31.9) [12–15]. Thus, we conclude that the double bond at C-29 in euglenatide B has been shifted by two carbons to C-31 in euglenatide B_2_ (Fig. 5a).

Similarly, our data suggest that euglenatides A_2_ and C_2_ are exactly the same as euglenatides A and C, but one of the double bonds in the triene has been shifted by two carbons (Fig. 5a). Specifically, our data show that (1) euglenatides A-C and A_2_-C_2_ have the same exact masses and MS/MS fragmentation patterns (Fig. 3, Fig. S1), (2) the conjugated triene chromophore in euglenatides A-C is absent in A_2_-C_2_ (Fig. 2b, c), and (3) the euglenatides and their analogs have regularly spaced elution times by LC–MS (Fig. 2b). Several trials were made to purify euglenatides A, A_2_, C and C_2_ for NMR analysis, but unfortunately their low abundance and co-elution with other metabolites hindered their purification. 

Previously, it was shown that euglenatide B had antiproliferative activity in the mid-nanomolar range against mammalian cancer cell lines, including A549 lung adenocarcinoma cells. To test the antiproliferative activity of the euglenatide analogs and thus the importance of the conjugated triene motif, we tested the activity of both euglenatides B and B_2_ against A549 cells (Fig. 5b). Our data confirm the potency of euglenatide B in the mid-nanomolar range, with an IC_50_ of 358 nM, in comparison to the previously reported IC_50_ of 773 nM. The data also show that euglenatide B_2_ is about tenfold less potent than euglenatide B, with an IC_50_ of 2987 nM. Thus, the conjugated triene is quite important for the activity of the euglenatides, and this information should inform future efforts to characterize the structure–activity relationships of the euglenatides.

## Conclusions

Here, we show that *E. gracilis* produces not only the previously reported euglenatides A–C, but also a related family of euglenatide analogs, euglenatides A_2_–C_2_, in roughly equal amounts. In the euglenatide analogs, one of the double bonds in the conjugated triene has been shifted by two carbons, thereby disrupting the chromophore. The euglenatide B_2_ analog was fully characterized by NMR spectroscopy and mass spectrometry and shown to have antiproliferative activity that is roughly tenfold less than euglenatide B against A549 lung adenocarcinoma cells, indicating that the conjugated triene is important to the activity of the euglenatides. This information helps to characterize the structure–activity relationships of the euglenatides.

## General experimental procedures

### Cultivation of *E. gracilis*

*E. gracilis var. saccharophila* (CCAP 1224/5T) was obtained from the Culture Collection of Algae and Protozoa (United Kingdom). An axenic stock was generated by plating the algae on plates containing a 1:1 mix of *E. gracilis* medium (EG) and Jaworski’s medium (JM) and 15 g/L of agar and picking a single colony. EG and JM were made using a previously described method [6]. *E. gracilis* was seeded typically in a 1:10 ratio into EG:JM medium in an Erlenmeyer flask and incubated at room temperature under a daylight lamp for 7d on a 150 rpm shaker. The lamp was set at a 12 h light/12 h dark cycle with an intensity of 2000 lumens.

### Large scale cultivation of *E. gracilis* for euglenatide production

The EG:JM culture of *E. gracilis* was adjusted to 4 g/L (Abs 0.1 at 740 nm). 75 mL of this suspension was harvested by centrifugation at 3000 g at 4 °C for 20 min, washed three times with sterile Milli-Q water to remove any trace components of EG:JM medium, and seeded into 1.5L of synthetic medium (JM + 1 g/L sodium acetate trihydrate + 0.1 g/L calcium chloride) with 30 mM Glu (pH adjusted to 4.8 prior to autoclaving) in each bioreactor. The culture was incubated at room temperature under a daylight lamp at 12 h light/12 h dark cycle for 10d before collecting the cells for further extraction. Approximately 12 L of culture was generated in all. The cultures were centrifuged at 3000 g for 20 min, and the culture medium was discarded.

### Purification of euglenatides

 12L worth of cells were extracted with 2L of 90% MeOH. The 90% MeOH was extracted with 2L of hexanes. The MeOH layer was evaporated to yield the methanolic extractives (1.6 g) that were further fractionated on a C18 reversed phase flash column (300 mm × 46 mm, 80 g silica) with a 10% (200 mL) step-wise gradient elution from 20 to 100% MeCN. HPLC–DAD-MS analysis revealed the fraction eluting with 60% MeCN to be rich in euglenatides (Fig. S2). This fraction (80 mg) was chromatographed on an LH-20 Sephadex gel column, eluting with MeOH. Based on HPLC–DAD-MS analysis, subfractions containing mainly metabolites of *m/z*(–) 808 (euglenatide B and its analog) were combined (25 mg) and subjected to repeated semi-preparative reversed phase HPLC (HS C18-10, 250 mm × 10 mm, 10 μm, 3 mL/min) with an isocratic elution over 35 min with 34% MeCN/H_2_O to yield euglenatide B (3 mg) and its new isomer, euglenatide B_2_ (3 mg) (Fig. S2).

### Euglenatide B

 White powder; NMR (600 MHz, DMSO-*d*_6_) see Table 1 and Table S1 and Figs. S3, S4; ESI(−)MS *m*/*z* 808 [M − H]^−^ and *m/z* 844 [M − H + Cl]^−^; HRESI(+)MS *m*/*z* 810.4618 [M + H]^+^ (calcd for C_38_H_64_N_7_O_12_, 810.4613).

### Euglenatide B_2_

 White powder; NMR (600 MHz, DMSO-*d*_6_) see Table 1 and Table S2 and Figures S5-S13; ESI(−)MS *m*/*z* 808 [M − H]^−^ and *m/z* 844 [M − H + Cl]^−^; HRESI(+)MS *m*/*z* 810.4617 [M + H]^+^ (calcd for C_38_H_64_N_7_O_12_, 810.4613).

### Antiproliferation assay

 A549 cells were cultured in DMEM medium supplemented with 10% FBS, 100 U/mL penicillin and 100 μg/μL streptomycin. 6 × 10^3^ A549 cells per 100 μL were seeded in a 96-well plate and incubated at 37° C with 5% CO_2_. After 22 h of incubation, each well was treated with 1 μL of dimethyl sulfoxide (DMSO) or 1 μL of euglenatide B or euglenatide B_2_ in DMSO at final concentration of 10^5^, 10^4^, 10^3^, 10^2^, 10, or 1 nM. Each concentration was tested in triplicate. The plate was incubated for 72 h, and cell viability was tested with the MTS assay (Promega). IC_50_ values were calculated using GraphPad. Wells with only culture media were used as a blank, and wells seeded with A549 cells and treated with 1% DMSO were used as a negative control. The viability of treated A549 cells relative to the negative control was calculated after a blank correction.

## Supplementary Information

Additional file 1.

## Data Availability

The authors will provide access to all datasets upon request.
