# Supplementary material for: Discovery of a parallel family of euglenatide analogs in Euglena gracilis
Source: Nat Prod Bioprospect. 2025 Jan 6;15(1):10. doi: 10.1007/s13659-024-00490-8 (PMC11703798; doi:10.1007/s13659-024-00490-8)
Supplement: Supplementary file 1 — Additional file 1. [file 13659_2024_490_MOESM1_ESM.pdf]

Supplementary Information

Discovery of a parallel family of euglenatide analogs in *Euglena gracilis*

Ahmed H. Elbanna,[1,2] Xinhui Kou,[1,†] Dilip V. Prajapati,[1,†] Surasree Rakshit,[1] and Rebecca A. Butcher[1,*]

[1]Department of Chemistry, University of Florida, Gainesville, Florida 32611, USA

[2]Department of Pharmacognosy, Faculty of Pharmacy, Cairo University, Cairo 11562, Egypt

[†]equal contributions

[*]Correspondence: butcher@chem.ufl.edu

Table of Contents

Figure S1. MS-MS fragmentation patterns of the euglenatides and their analogs. 3

Figure S2. Isolation scheme of euglenatides. 4

Table S1. Comparison of NMR data for euglenatide B and previously reported data. 5

Figure S3. ^{1}H NMR (600 MHz, DMSO-d_6) spectrum for euglenatide B. 7

Figure S4. ^{13}C NMR (150 MHz, DMSO-d_6) spectrum for euglenatide B. 7

Table S2. 1D and 2D NMR (600 MHz, DMSO-d_6) data for euglenatide B_2. 8

Figure S5. ^{1}H NMR (600 MHz, DMSO-d_6) spectrum for euglenatide B_2. 10

Figure S6. ^{13}C NMR (150 MHz, DMSO-d_6) spectrum for euglenatide B_2. 10

Figure S7. Comparison of ^{1}H NMR spectra for euglenatide B and B_2. 11

Figure S8. Comparison of expanded upfield region of ^{13}C NMR spectra for euglenatide B and B_2. 11

Figure S9. Comparison of expanded downfield region of ^{13}C NMR spectra for euglenatide B and B_2. 12

Figure S10. DQF-COSY NMR (600 MHz, DMSO-d_6) spectrum for euglenatide B_2. 12

Figure S11. Expanded DQF-COSY NMR (600 MHz, DMSO-d_6) spectrum for euglenatide B_2. 13

Figure S12. HSQC NMR (600 MHz, DMSO-d_6) spectrum for euglenatide B_2. 14

Figure S13. HMBC NMR (600 MHz, DMSO-d_6) spectrum for euglenatide B_2. 15

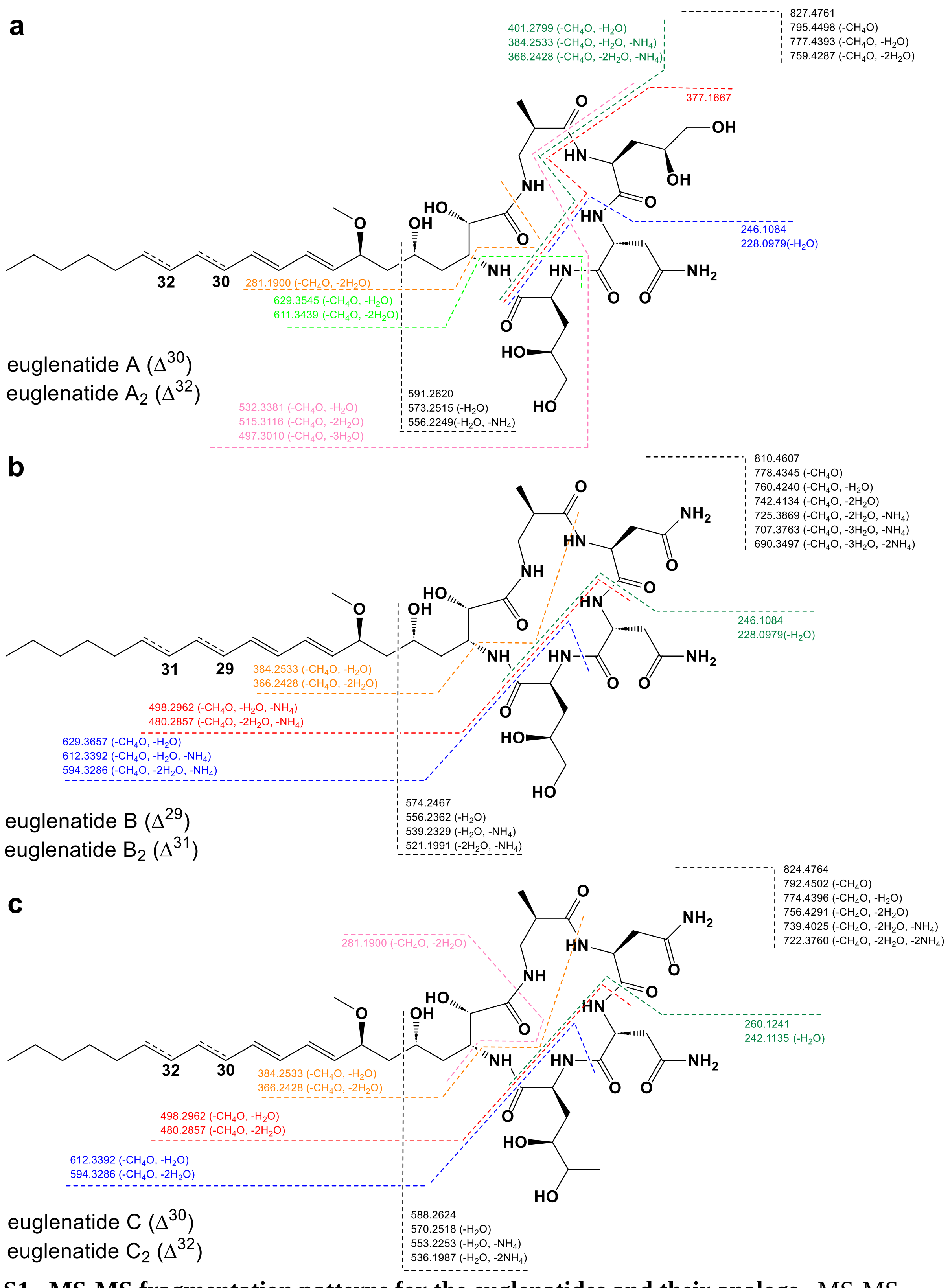

Figure S1. MS-MS fragmentation patterns for the euglenatides and their analogs. MS-MS fragmentation patterns for euglenatides A and A$_2$ (**a**), euglenatides B and B$_2$ (**b**), and euglenatides C and C$_2$ (**c**).

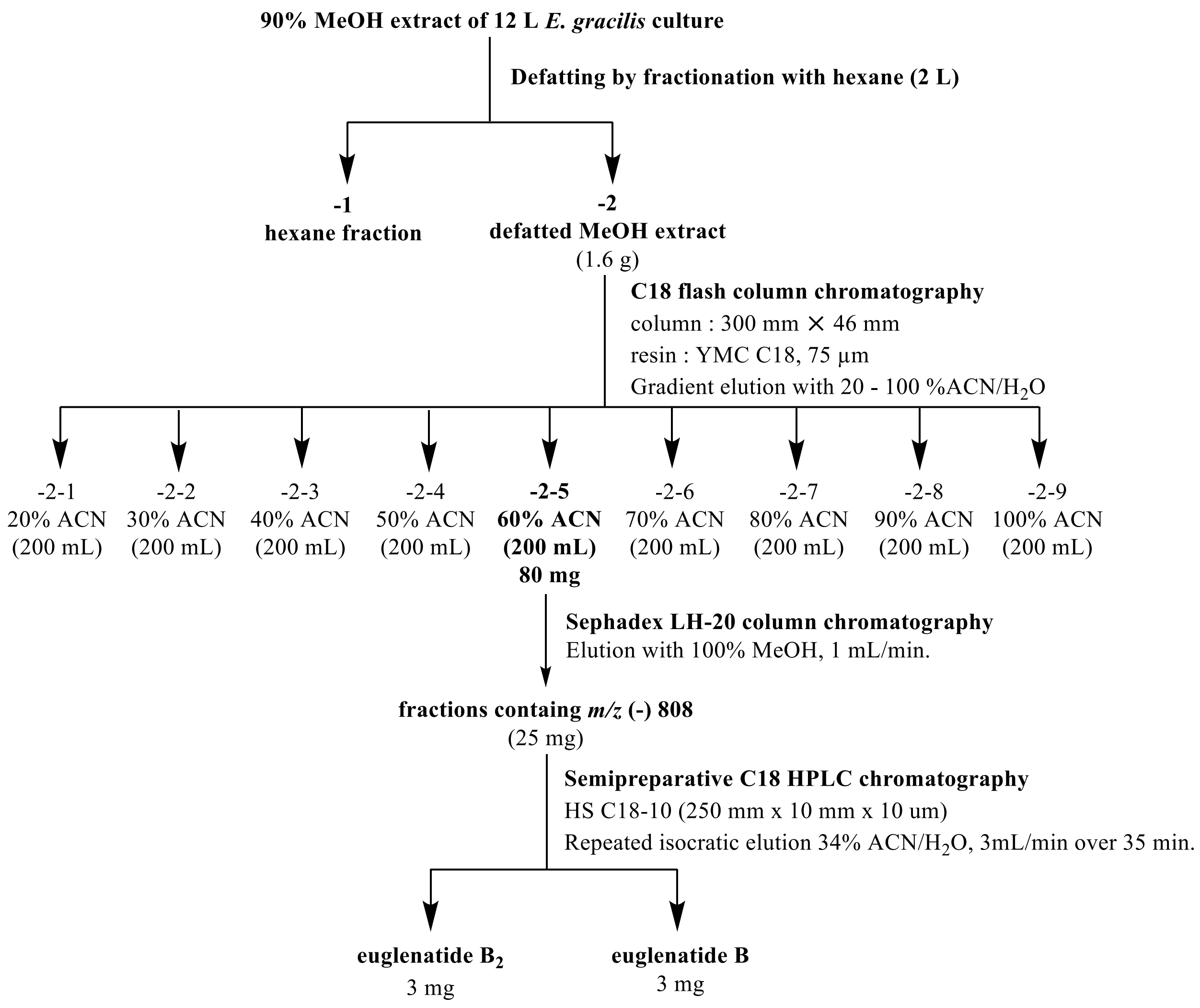

Figure S2. Isolation scheme of euglenatides.

Table S1. Comparison of NMR data for euglenatide B and previously reported data. 1D and 2D NMR (600 MHz, DMSO-d_6) data for euglenatide B are compared to previously reported data for euglenatide B.

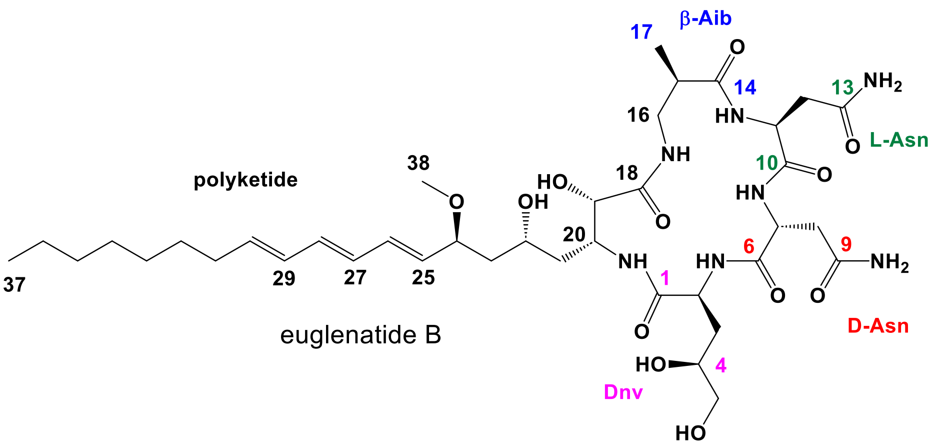

No.	euglenatide B (data reported here)			euglenatide B (previously reported data)		
	Type	^{13}C ppm	^{1}H ppm mult. (*J* in Hz)	Type	^{13}C ppm	^{1}H ppm mult. (*J* in Hz)
**Dnv**						
**1**	C	172.5	---	C	172.4	---
**2**	CH	50.3	4.30, ddd (*11.0, 7.8, 2.8*)	CH	50.3	4.32, m
**2-NH**	NH	---	7.65, d (7.8)	NH	---	7.65, d (*7.8*)
**3**	CH_2	34.3	1.82, ddd (*14.0, 11.0, 2.8*)	CH_2	34.3	1.86, m
			1.70, m			1.71, m
**4**	CH	67.5	3.44, m	CH	67.5	3.43, m
**4-OH**	OH	---	4.49, d (*5.0*)	OH	---	4.44, br d (*4.7*)
**5**	CH_2	66.3	3.31, m	CH_2	66.3	3.32, m
			3.24, m			3.26, m
**5-OH**	OH	---	4.39, m	OH	---	4.38, m
**D-Asn**						
**6**	C	171.2	---	C	171.1	---
**7**	CH	49.7	4.51, m	CH	49.3	4.45, m
**7-NH**	NH	---	8.20, br d (*7.8*)	NH		7.77, d (*7.9*)
**8**	CH_2	36.2	2.93, dd (*16.0, 7.0*)	CH_2	35.8	2.94, dd (*16.8, 5.0*)
			2.56, dd (*16.0, 3.6*)			2.57, dd (*16.9, 3.7*)
**9**	C	173.1	---	C	173.4	---
**9-NH**	NH_2	---	7.87, s	NH_2	---	7.78 s
			7.14, s			7.29, s
**L-Asn**						
**10**	C	169.7	---	C	169.6	---
**11**	CH	51.6	4.04, m	CH	51.6	4.06. m
**11-NH**	NH	---	9.05, d (*6.4*)	NH	---	9.02, d (*6.5*)
**12**	CH_2	35.2	2.86, dd (*15.8, 4.0*)	CH_2	35.0	2.86, dd (*15.7, 4.0*)
			2.43, dd (*15.8, 9.3*)			2.47, dd (*15.9, 4.7*)
**13**	C	172.2	---	C	172.1	---
**13-NH**	NH_2	---	7.37, s	NH_2	---	7.36, s
			6.86, s			6.88, s

No.	euglenatide B (data reported here)			euglenatide B (previously reported data)		
	Type	^{13}C ppm	^{1}H ppm mult. (*J* in Hz)	Type	^{13}C ppm	^{1}H ppm mult. (*J* in Hz)
**β-Aib**						
**14**	C	176.4	---	C	176.3	---
**15**	CH	37.6	2.66, m	CH	37.5	2.66, m
**16**	CH_2	41.7	3.23, m	CH_2	41.7	3.27, m
			2.99, br d (*13.2*)			3.03, br d (*13.0*)
**16-NH**	NH	---	7.72, br s	NH	---	7.06, s
**17**	CH_3	17.2	1.03, d (*7.0*)	CH_3	16.8	1.04, d (*7.1*)
**Polyketide**						
**18**	C	172.1	---	C	172.3	---
**19**	CH	72.6	3.89, m	CH	72.9	3.86, dd (*9.6, 7.7*)
**19-OH**	OH	---	5.10, br s	OH	---	5.13, br d (*7.7*)
**20**	CH	49.1	3.88, m	CH	49.1	3.92, m
**20-NH**	NH	---	6.93, d (*9.1*)	NH	---	6.90, d (*9.6*)
**21**	CH_2	40.5	1.69, m	CH_2	40.3	1.69, m
			1.19, m			1.20, m
**22**	CH	62.9	3.49, m	CH	63.0	3.47, m
**22-OH**	OH	---	4.38, m	OH	---	4.31, m
**23**	CH_2	43.7	1.47, m	CH_2	43.8	1.49, m
			1.33, m			1.30, br dd (*14.5, 5.1*)
**24**	CH	77.8	3.72, ddd (*11.5, 7.6, 3.7*)	CH	77.8	3.73, m
**25**	CH	133.9	5.50, dd (*14.6, 7.6*)	CH	133.9	5.51, dd (*14.6, 7.6*)
**26**	CH	131.2	6.19, dd (*14.6, 10.4*)	CH	131.3	6.20, dd (*14.6, 10.3*)
**27**	CH	130.0	6.14, dd (*14.6, 10.4*)	CH	130.0	6.15, dd (*14.6, 10.3*)
**28**	CH	132.8	6.23, dd (*14.5, 10.4*)	CH	132.8	6.24, dd (*14.5, 10.4*)
**29**	CH	130.3	6.06, dd (*15.0, 10.4*)	CH	130.3	6.07, dd (*15.1, 10.4*)
**30**	CH	135.1	5.71, dt (*15.0, 7.1*)	CH	135.1	5.72, dt (*15.1, 7.1*)
**31**	CH_2	32.1	2.06, q (*7.0*)	CH_2	32.1	2.06, q (*7.0*)
**32**	CH_2	28.7	1.34, m	CH_2	28.7	1.35, m
**33**	CH_2	28.50	1.25, m	CH_2	28.52	1.25, m
**34**	CH_2	28.52	1.25, m	CH_2	28.54	1.25, m
**35**	CH_2	31.2	1.24, m	CH_2	31.2	1.24, m
**36**	CH_2	22.0	1.26, m	CH_2	22.0	1.25, m
**37**	CH_3	13.9	0.85, t (*7.0*)	CH_3	13.9	0.85, t (*6.8*)
**38**	OCH_3	55.5	3.13, s	OCH_3	55.5	3.13, s

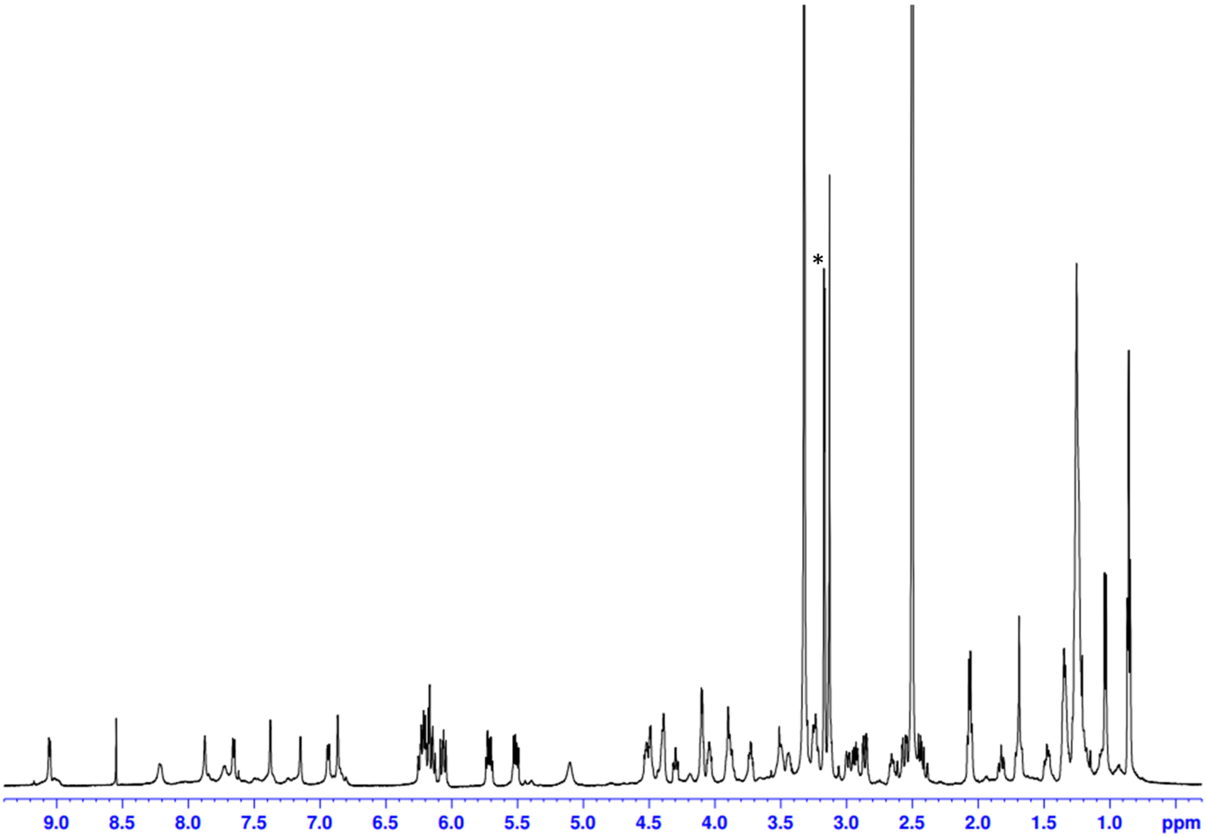

Figure S3. ^{1}H NMR (600 MHz, DMSO-*d*$_6$) spectrum for euglenatide B. *Residual methanol solvent.

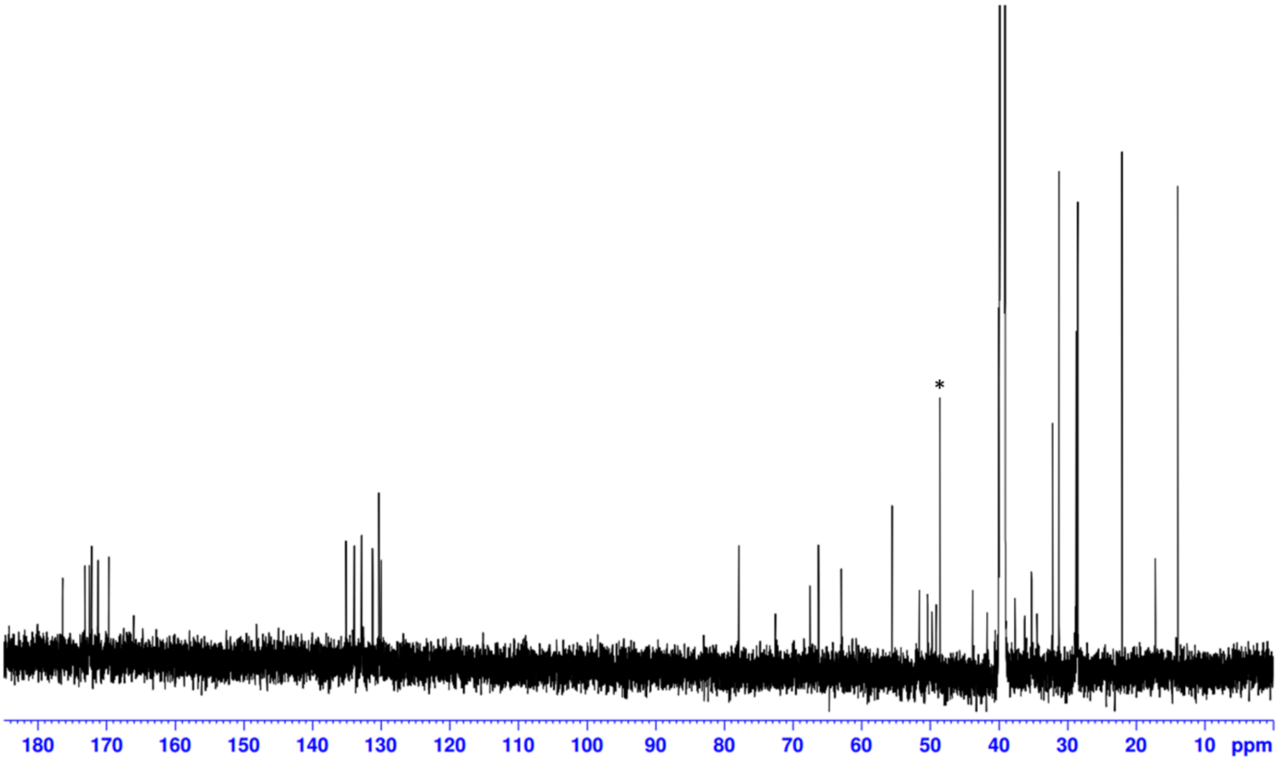

Figure S4. ^{13}C NMR (150 MHz, DMSO-*d*$_6$) spectrum for euglenatide B. *Residual methanol solvent.

Table S2. 1D and 2D NMR (600 MHz, DMSO-d_6) data for euglenatide B_2.

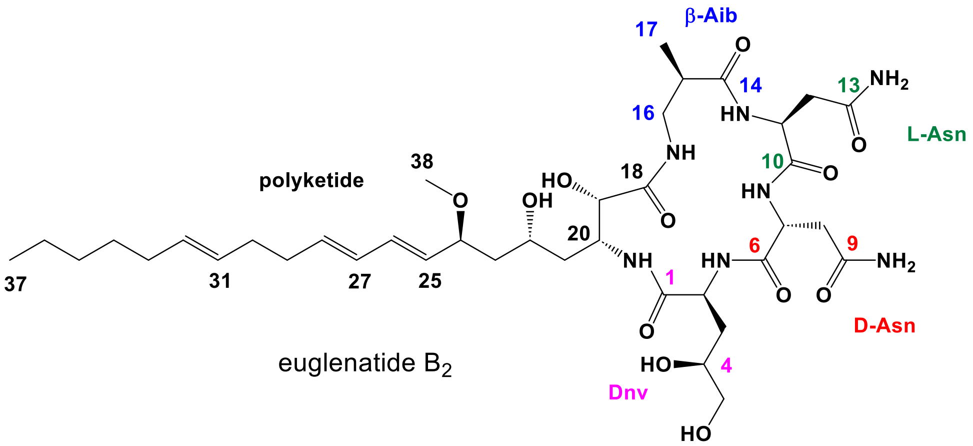

No.	Type	^{13}C ppm	^{1}H ppm mult. (*J* in Hz)	COSY	HMBC
			**Dnv**		
**1**	C	172.5	---	---	---
**2**	CH	50.3	4.29, ddd (*11.0, 7.8, 2.8*)	2-NH, 3	
**2-NH**	NH	---	7.65, d (*8.0*)	2	6
**3**	CH_2	34.4	1.82, ddd (*13.8, 10.7, 2.8*)	2, 4	
			1.69, m	2, 4	
**4**	CH	67.5	3.43, m	3	
**4-OH**	OH	---	4.48, d (*5.0*)		
**5**	CH_2	66.3	3.31, m		
			3.24, m		
**5-OH**	OH	---	4.39, m		
			**D-Asn**		
**6**	C	171.2	---	---	---
**7**	CH	49.7	4.50, m	7-NH, 8	
**7-NH**	NH	---	8.13, br d (*7.4*)	7	
**8**	CH_2	36.2	2.92, dd (*16.0, 6.8*)	7	6, 7, 9
			2.55, dd (*16.0, 3.7*)	7	6, 7, 9
**9**	C	173.2	---		
**9-NH**	NH_2	---	7.88, s		
			7.17, s		
			**L-Asn**		
**10**	C	169.7	---	---	---
**11**	CH	51.6	4.04, ddd (*9.8, 6.4, 4.1*)	11-NH, 12	
**11-NH**	NH	---	9.04, d (*6.7*)	11	10, 11, 14
**12**	CH_2	35.2	2.85, dd (*15.6, 4.0*)	11	10, 11, 13
			2.43, dd (*15.6, 9.3*)	11	10, 11, 13
**13**	C	172.1	---	---	---
**13-NH**	NH_2	---	7.37, s		12
			6.86, s		
			**β-Aib**		

No.	Type	^{13}C ppm	^{1}H ppm mult. (*J* in Hz)	COSY	HMBC
**14**	C	176.4	---	---	---
**15**	CH	37.6	2.65, (9*.8*, *7.1*, *2.6*)	16, 17	
**16**	CH_2	41.7	3.23, m	16-NH, 15	18
			2.99, br d (*13.2*)	16-NH, 15	18
**16-NH**	NH	---	7.61, br s	16	
**17**	CH_3	17.1	1.03, d (*7.0*)	15	14, 15, 16
**Polyketide**					
**18**	C	172.2	---	---	---
**19**	CH	72.6	3.89, m		20
**19-OH**	OH	---	5.10, very broad		
**20**	CH	49.1	3.88, m	20-NH, 21	
**20-NH**	NH	---	6.92, d (*8.9*)	20	
**21**	CH_2	40.5	1.68, m	20, 22	
			1.18, m	20, 22	
**22**	CH	62.9	3.49, m	21, 23	
**22-OH**	OH	---	4.36, m		
**23**	CH_2	43.8	1.46, m	22, 24	
			1.31, m	22, 24	
**24**	CH	77.8	3.7, ddd (*11.5*, *8.1*, *3.5*)	23, 25	
**25**	CH	132.2	5.40, m	24, 26	
**26**	CH	131.3	6.12, dd (*15.0*, *10.4*)	25, 27	24, 28
**27**	CH	129.9	6.04, dd (*15.0*, *10.4*)	26, 28	25, 29
**28**	CH	133.8	5.69, dt (*14.5*, *6.5*)	27, 29	26, 29, 30
**29**	CH_2	32.2	2.10, m	28, 30	27, 28, 31
**30**	CH_2	31.7	2.04, m	29, 31	31, 32
**31**	CH	129.3	5.38, dd (*16.2*, *5.4*) [a]	30	30, 33
**32**	CH	130.5	5.40, dd (*16.2*, *5.4*) [a]	33	30, 33, 34
**33**	CH_2	31.2	1.93, q (*6.1*)		31, 32, 34, 35
**34**	CH_2	28.6	1.30, m		32, 33, 35, 36
**35**	CH_2	30.7	1.22, m		36
**36**	CH_2	21.9	1.25, m	37	
**37**	CH_3	13.9	0.85, t (*7.0*)	36	35, 36
**38**	OCH_3	55.5	3.11, s		24

[a] *J* values were obtained from DQF-COSY data.

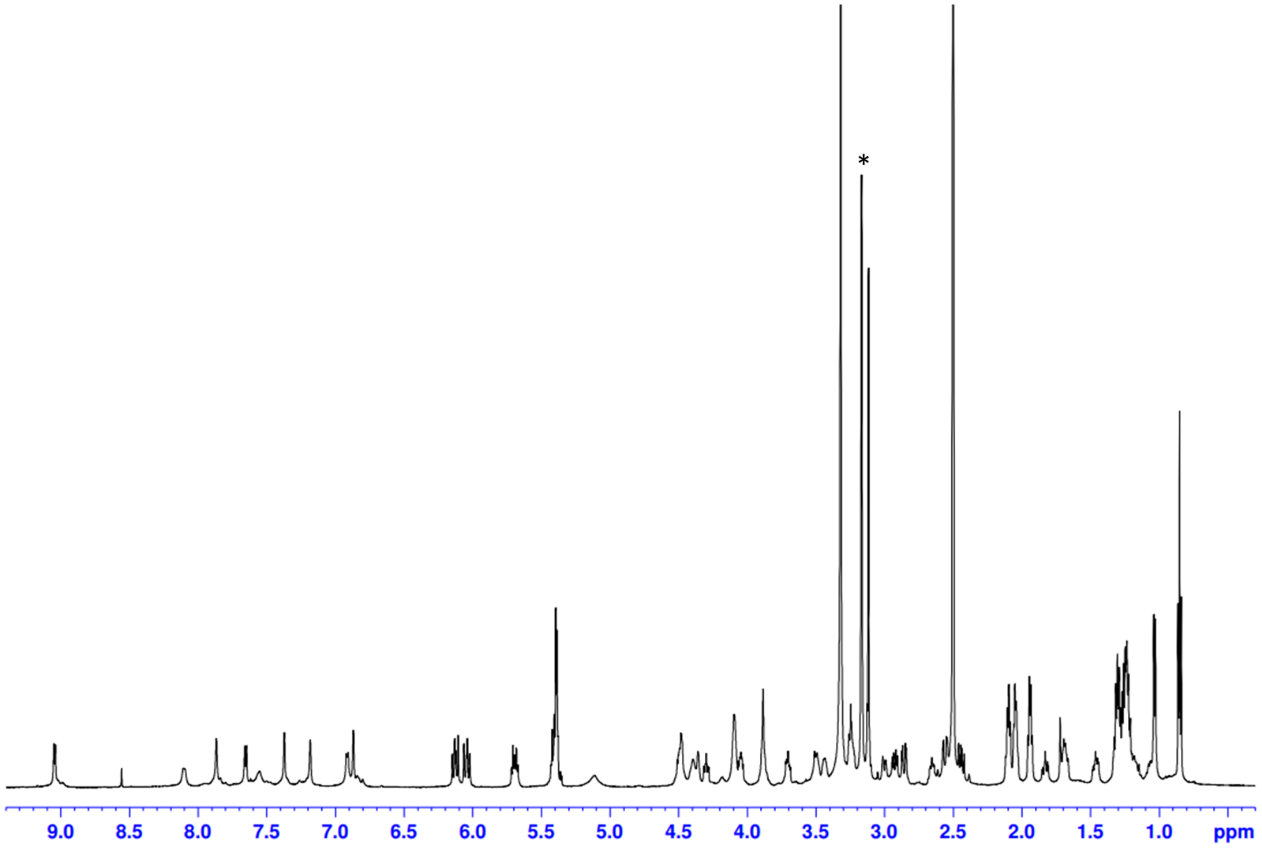

Figure S5. ^{1}H NMR (600 MHz, DMSO-*d*$_6$) spectrum for euglenatide B$_2$. * Residual methanol solvent.

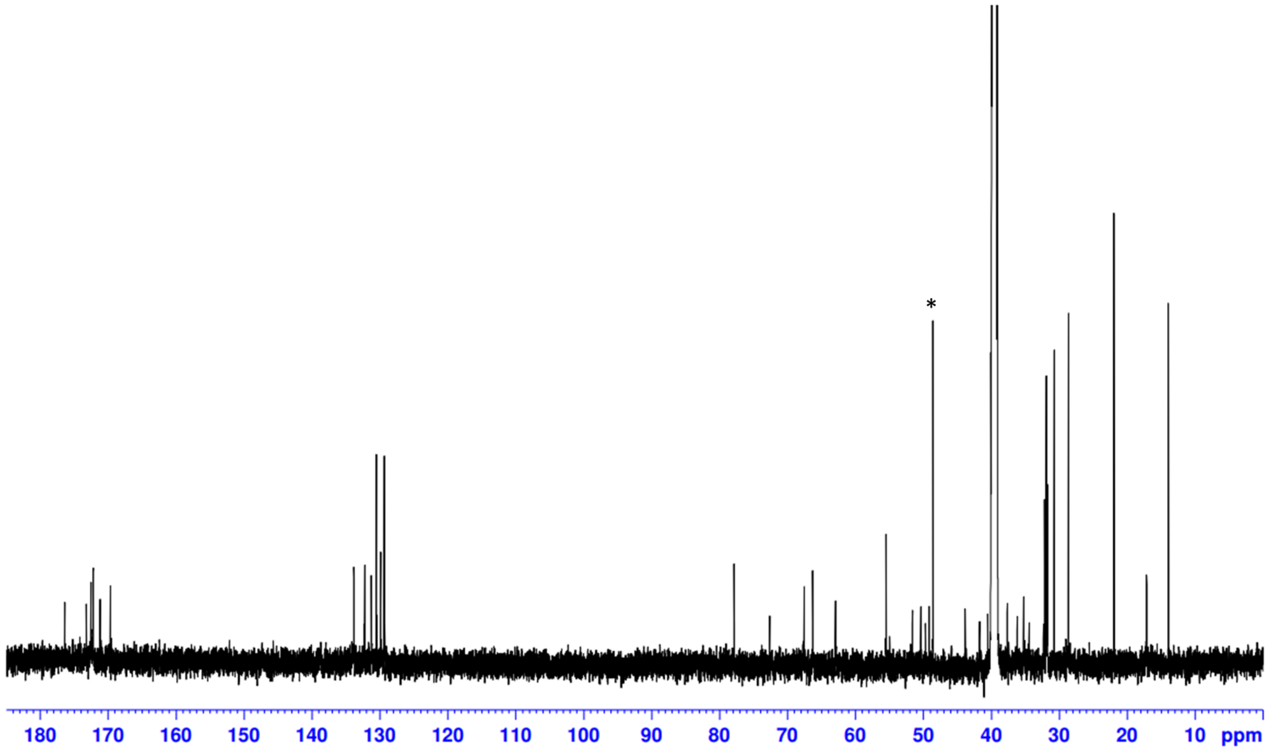

Figure S6. ^{13}C NMR (150 MHz, DMSO-*d*$_6$) spectrum for euglenatide B$_2$. * Residual methanol solvent.

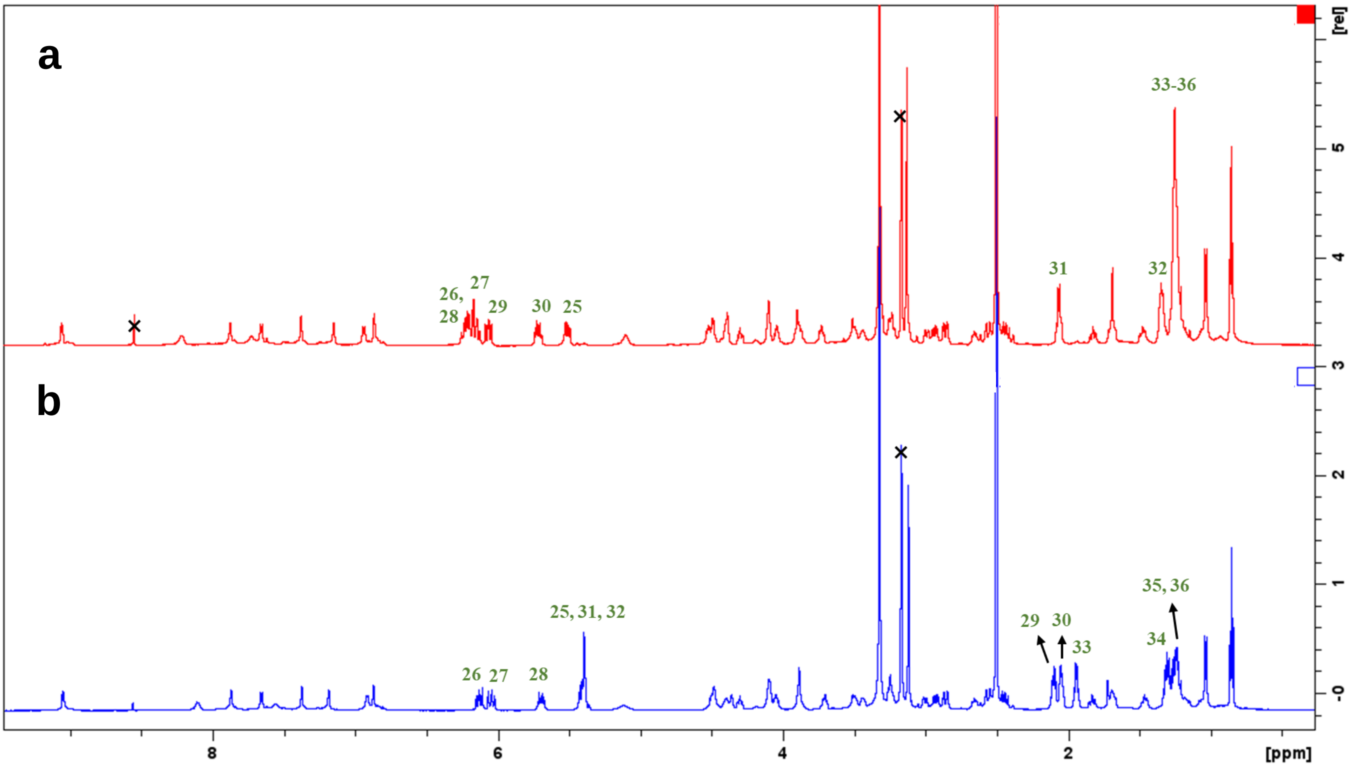

Figure S7. Comparison of ^{1}H NMR spectra for euglenatide B and B$_2$. ^{1}H NMR spectra (600 MHz, DMSO-d_6) spectra are shown for (**a**) euglenatide B against (**b**) euglenatide B$_2$ with main different signals annotated.

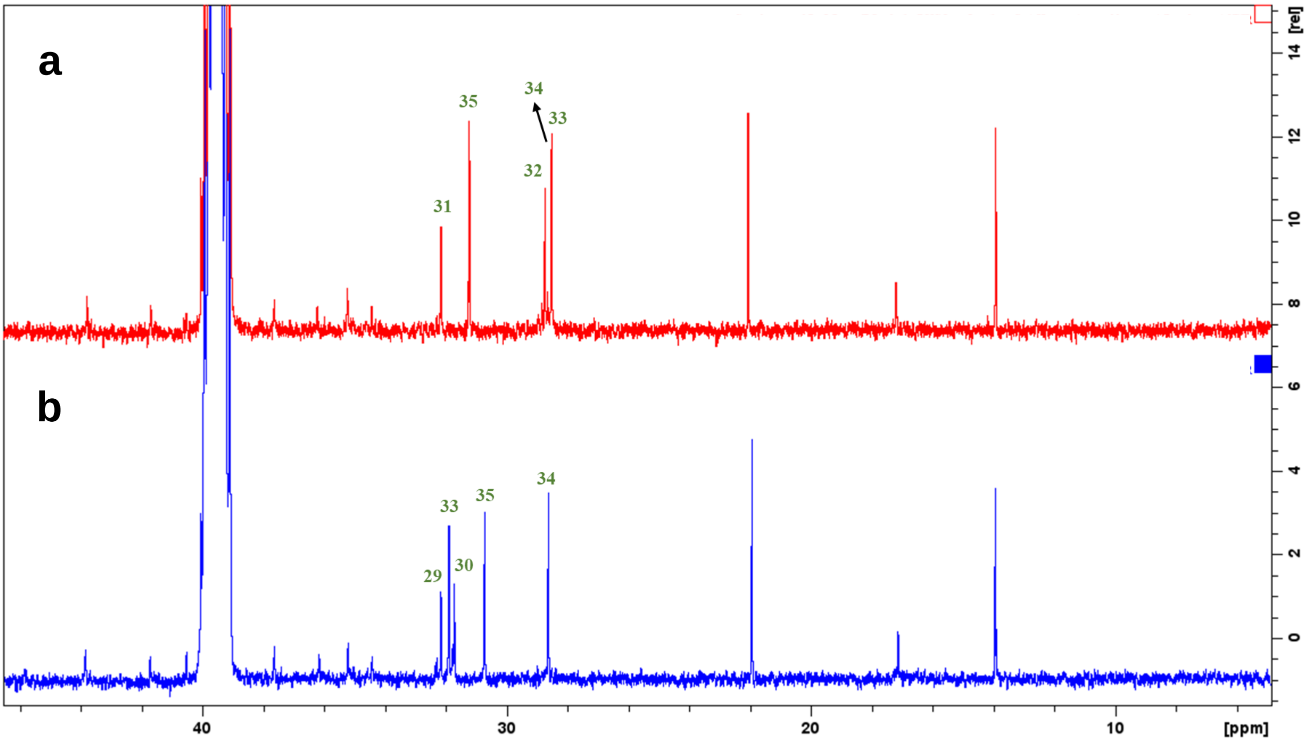

Figure S8. Comparison of expanded upfield region of ^{13}C NMR spectra for euglenatide B and B$_2$. An expanded view of the upfield region of the ^{13}C NMR (150 MHz, DMSO-d_6) spectra are shown for (**a**) euglenatide B against (**b**) euglenatide B$_2$, with main different signals annotated.

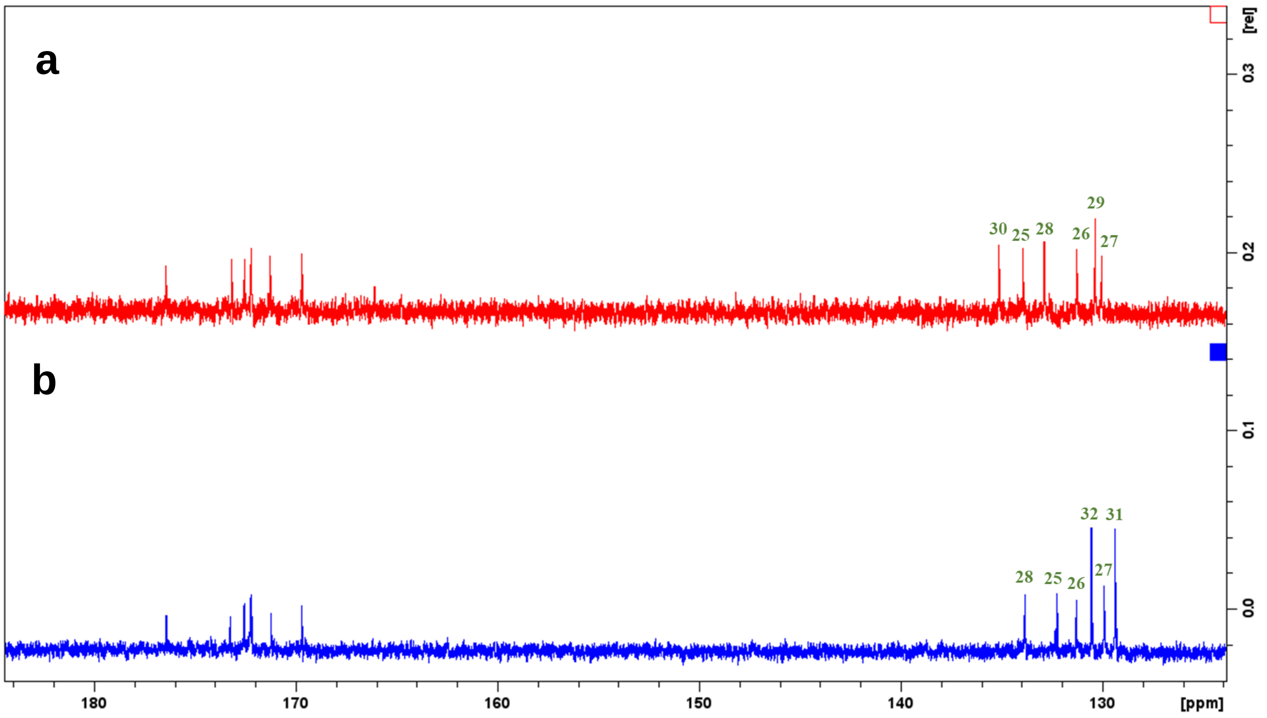

Figure S9. Comparison of expanded downfield region of ^{13}C NMR spectra for euglenatide B and B_2. An expanded view of the downfield region of the ^{13}C NMR (150 MHz, DMSO-d_6) spectra are shown for (**a**) euglenatide B against (**b**) euglenatide B_2, with main different signals annotated.

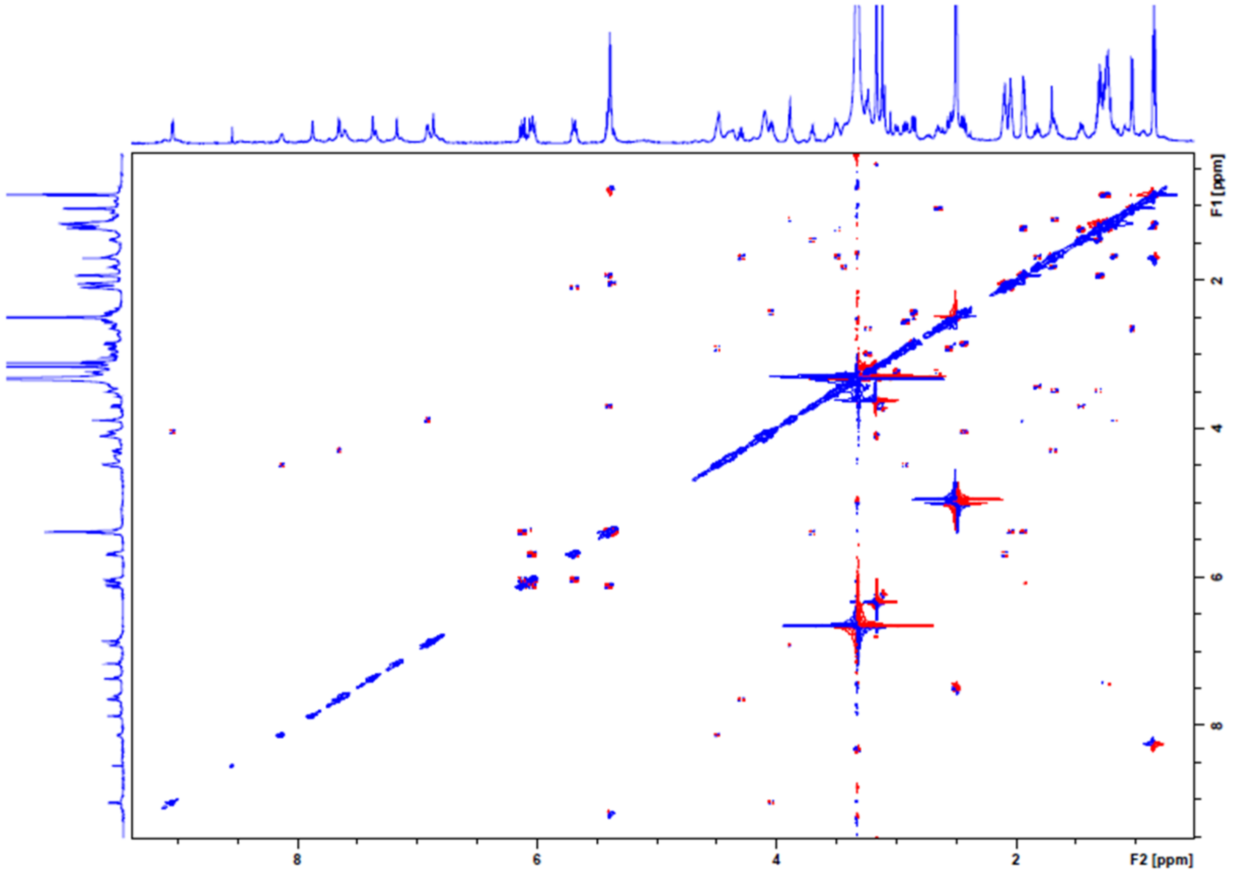

Figure S10. DQF-COSY NMR (600 MHz, DMSO-d_6) spectrum for euglenatide B_2.

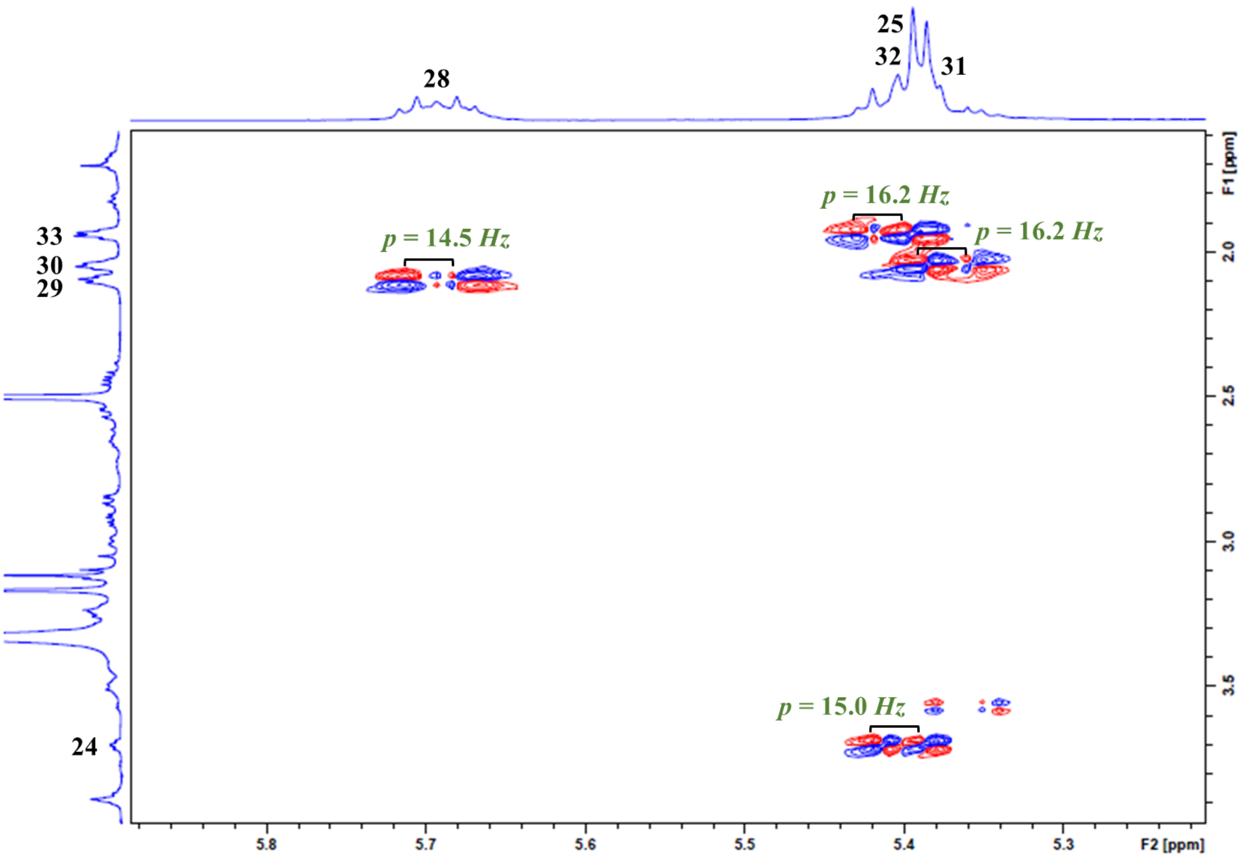

Figure S11. Expanded DQF-COSY NMR (600 MHz, DMSO-d_6) spectrum for euglenatide B_2. *p* = passive coupling constants.

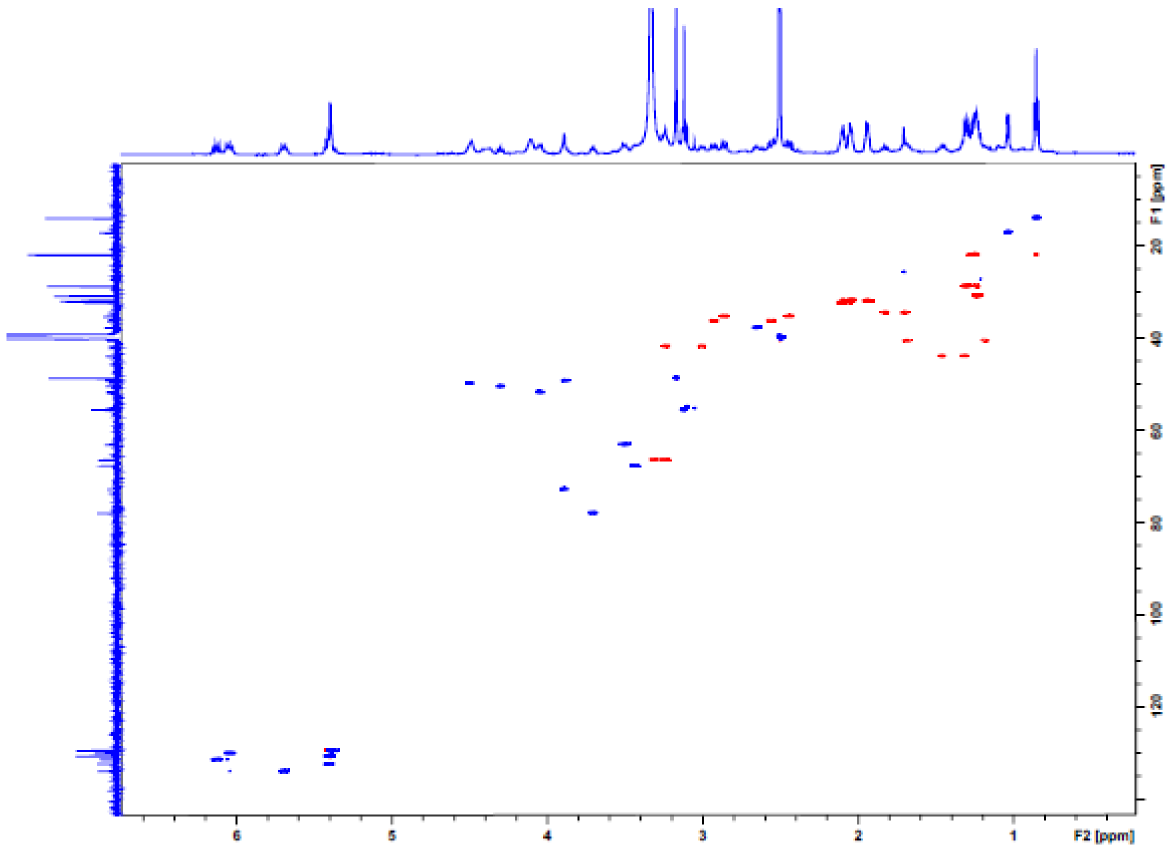

Figure S12. HSQC NMR (600 MHz, DMSO-d_6) spectrum for euglenatide B_2.

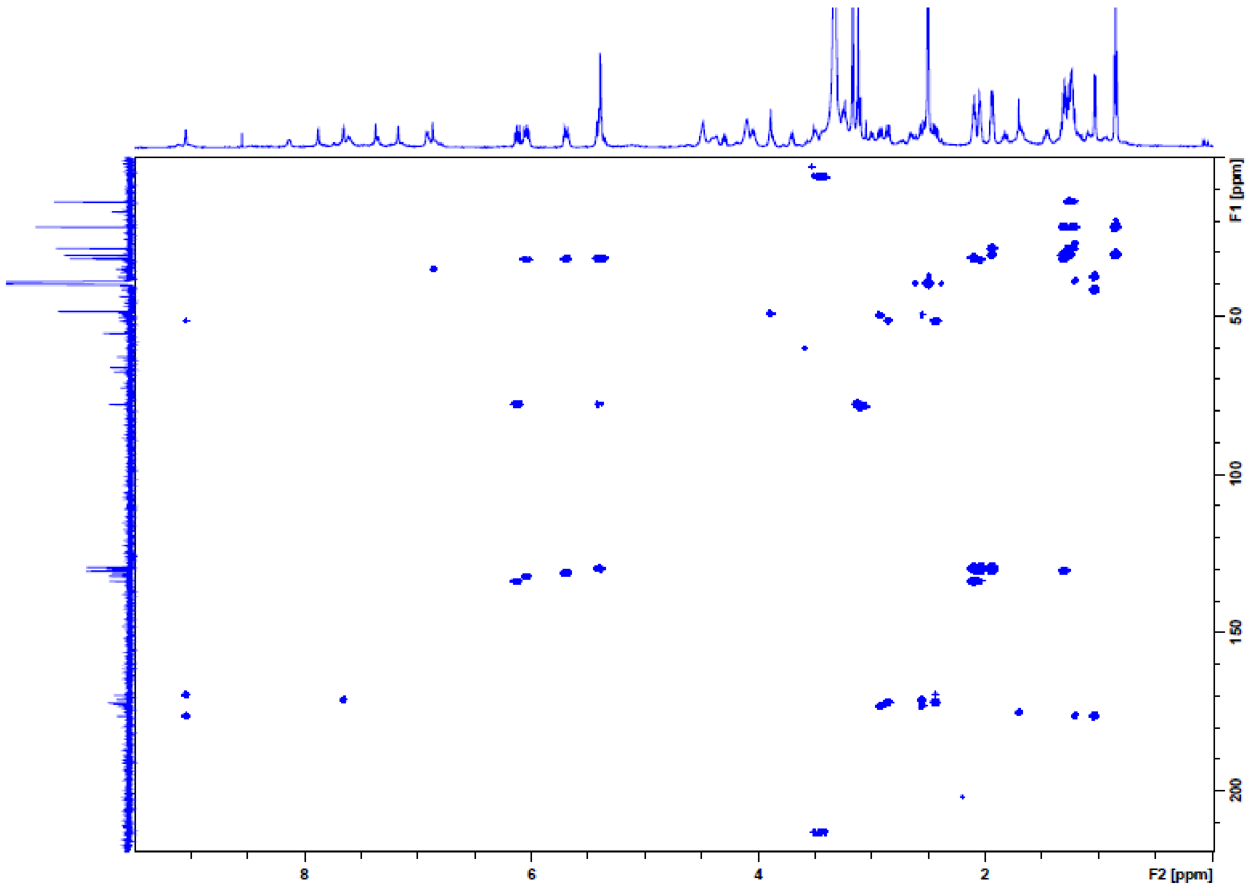

Figure S13. HMBC NMR (600 MHz, DMSO-*d*$_6$) spectrum for euglenatide B$_2$.
